# Neuroligin-1 is altered in the hippocampus of Alzheimer’s disease patients and mouse models, and modulates the toxicity of amyloid-beta oligomers

**DOI:** 10.1038/s41598-020-63255-6

**Published:** 2020-04-24

**Authors:** Julien Dufort-Gervais, Chloé Provost, Laurence Charbonneau, Christopher M. Norris, Frédéric Calon, Valérie Mongrain, Jonathan Brouillette

**Affiliations:** 1 Department of Pharmacology and Physiology, Université de Montréal, Montréal, Québec, Canada; 2 Center for Advanced Research in Sleep Medicine, Hôpital du Sacré-Coeur de Montréal (Recherche CIUSSS-NIM), Montréal, Québec, Canada; 3 Department of Neuroscience, Université de Montréal, Montréal, Québec, Canada; 4 Department of Molecular and Biomedical Pharmacology, Sanders-Brown Center on Aging, University of Kentucky, Lexington, KY USA; 5 Neuroscience Unit, Research Center - CHU de Québec, Québec, QC Canada; 6 Faculty of Pharmacy, Université Laval, Québec, QC Canada

**Keywords:** Molecular neuroscience, Ageing, Diagnostic markers

## Abstract

Synapse loss occurs early and correlates with cognitive decline in Alzheimer’s disease (AD). Synaptotoxicity is driven, at least in part, by amyloid-beta oligomers (Aβo), but the exact synaptic components targeted by Aβo remain to be identified. We here tested the hypotheses that the post-synaptic protein Neuroligin-1 (NLGN1) is affected early in the process of neurodegeneration in the hippocampus, and specifically by Aβo, and that it can modulate Aβo toxicity. We found that hippocampal NLGN1 was decreased in patients with AD in comparison to patients with mild cognitive impairment and control subjects. Female 3xTg-AD mice also showed a decreased NLGN1 level in the hippocampus at an early age (i.e., 4 months). We observed that chronic hippocampal Aβo injections initially increased the expression of one specific *Nlgn1* transcript, which was followed by a clear decrease. Lastly, the absence of NLGN1 decreased neuronal counts in the dentate gyrus, which was not the case in wild-type animals, and worsens impairment in spatial learning following chronic hippocampal Aβo injections. Our findings support that NLGN1 is impacted early during neurodegenerative processes, and that Aβo contributes to this effect. Moreover, our results suggest that the presence of NLGN1 favors the cognitive prognosis during Aβo-driven neurodegeneration.

## Introduction

Synapse loss is an early event in the pathogenesis of Alzheimer’s disease (AD) and correlates with cognitive decline of the patients^1–3^. Hippocampus-dependent memory for recent facts and events (explicit memory) is among the first type of memory that is affected in the disease because of the neurodegenerative process that rapidly and gradually takes place in the hippocampus at the onset of AD^4,5^. More precisely, within the hippocampal network, the perforant path that connects the entorhinal cortex to the dentate gyrus (DG) is one of the earliest and most severely affected pathways in AD^6,7^. This suggests that the DG is among sites showing initial signs of synaptic dysfunction, including in glutamatergic transmission^8^, and that it could represent an area of particular relevance for early interventions.

Despite decades of research focused on the amyloid-beta (Aβ) peptide, its causal role in AD pathogenesis remains unclear at the molecular level. However, strong evidence suggests that soluble Aβ oligomers (Aβo) are particularly neurotoxic and that their presence can correlate with memory deficits in AD patients and animal models, especially when considering oligomers derived from Aβ_1-42_ peptides^9–18^. Soluble Aβo start to accumulate in the human brain ~10 to 15 years before clinical symptoms and were shown to induce losses of excitatory synapses and neurons^19–22^. The oligomerization of Aβ is rapid and it has been suggested that Aβ toxicity results from multiple small Aβ species present at the same time, rather than from one particular type of soluble oligomer^11,18,23,24^. Although the toxicity (including synaptotoxicity) of soluble Aβo is well established, specific synaptic components that are altered when Aβo begin to increase in the brain at the onset of AD remain to be identified. Identifying Aβo-driven changes in proteins specifically involved in excitatory synapse functioning is required to understand glutamatergic synapse dysfunction and loss, which likely takes place before neurodegeneration induces substantial and irreversible brain damage permanently affecting cognition and autonomy.

Neuroligins (NLGNs) are post-synaptic adhesion proteins that interact with pre-synaptic protein neurexins (NRXNs) and have roles in synapse formation, maturation, maintenance and plasticity^25–27^. Neuroligin-1 (NLGN1) predominantly localizes at excitatory post-synaptic densities^28^, and previous work has linked it to neuropsychiatric and neurological disorders such as autism, schizophrenia and stroke^29–31^. Interestingly, NLGN1 has also been shown to be involved in synaptic plasticity, N-methyl-D-aspartate (NMDA) receptor function, memory and sleep regulation^25,32,33^, which are all altered in AD. The relevance of NLGN1 (and NRXNs) in the context of AD neurodegeneration has been emphasized in the last decade^3,34,35^. In fact, a single dose of Aβ_1-40_ fibrils has been shown to negatively impact NLGN1 in rats, which impaired synaptic function and memory^36^. In addition, Aβo were shown to bind to NLGN1 *in vitro*, and interfering with this interaction was observed to modulate synaptic integrity^37,38^. Moreover, NLGN1 was recently shown to be decreased in the plasma of patients with AD as well as in the preclinical period^39^. Nonetheless, to the best of our knowledge, it remains to be determined whether NLGN1 is altered in the hippocampus of AD patients as well as whether it is involved in the specific hippocampal pathology induced *in vivo* by soluble low-molecular-weight Aβo_1-42_.

Therefore, we here aimed to fill this knowledge gap using quantifications of the NLGN1 level in the hippocampus of patients with AD as well as in two animal models with Aβ-driven neurodegeneration. Importantly, we assessed the time course of the effect on NLGN1 by performing quantifications also in patients with amnesic mild cognitive impairment (aMCI), in triple transgenic (3xTg-AD) mice of 4, 12 and 18 months, and in mice submitted to 2, 4 and 6 days of Aβo_1-42_ injection in the hippocampus. In addition, we tested whether the absence of NLGN1 aggravates memory impairment and neuronal losses caused by Aβo_1-42_ using chronic hippocampal Aβo_1-42_ injections combined to immunohistochemistry and assessments of spatial and working memory. We found that the level of NLGN1 is decreased in the hippocampus of aMCI and AD patients and in young 3xTg-AD female mice, and that hippocampal Aβo_1-42_ injections decreased neuronal count in the DG and induced spatial learning deficits predominantly in *Nlgn1* knockout (KO) mice. Our results provide support to the hypothesis that NLGN1 is impacted early during Aβ pathology and that it modulates cognitive functions during Aβo-driven neurodegeneration.

## Methods

### Human brain tissues

Hippocampal protein samples from individuals with aMCI, AD patients and age-matched non-demented control subjects (CTRL) were provided by the brain bank of the Alzheimer’s Disease Center of the University of Kentucky^40^. AD and aMCI were diagnosed using clinical evaluations as previously described^40^. Briefly, cognitive status, neurologic and physical examinations were performed annually or biannually with a follow up of at least 2 years before death. All subjects had no comorbidity with substance abuse, head injury, encephalitis, meningitis, epilepsy, stroke, infectious disease or major psychiatric illness. Mini-mental state examination (MMSE) score was used as an indicator of overall cognitive status^41^, with a lower score being indicative of deficits in memory, attention, orientation and/or language. MMSE score was on average 24.4 and 7.8 in aMCI and AD patients, respectively (Table 1). Cognitive state was also evaluated with the animal naming test (ANIMALS: number of animals named in 1 min, with 12 generally considered as the cutoff for impairment), the Boston naming test (BNT: 15-item version with lower score indicating deficits), and the controlled oral word association test (COWA: sum of three trials of verbal fluency, lower score indicating impairment; Table 1). CTRL subjects were at Braak stage 0 or 1 and scored on average 27.8 on the MMSE (Table 1). Subjects were selected based on the shortest *post-mortem* interval (PMI) available to avoid protein degradation (Table 1). Other characteristics of patients and subjects are also listed in Table 1. Protocols for subjects and patients examinations and for the use of postmortem human brain tissue were approved by the University of Kentucky Institutional Review Board, and informed consent was obtained from all participants. All methods were performed in accordance with relevant guidelines and regulations.

**Table 1:** Characteristics of humans from which brain samples were collected.

Characteristics	Units	CTRL	aMCI	AD
Age	years	86.9 ± 1.7	89.5 ± 1.6	86.1 ± 1.2
Sexe	♀ (*n*)	7	5	6
	♂ (*n*)	5	6	7
PMI	hours	3.1 ± 0.1	3.7 ± 0.7	3.3 ± 0.2
NFTs	#/mm^2^	2.5 ± 1.4^a^	12.2 ± 4.0	33.3 ± 6.9^a^
Hippocampal soluble Aβ	ρmol/g	522.0 ± 135.1^b^	2006.8 ± 733.4^c^	5763.9 ± 1364.3^d^
MMSE	score	27.8 ± 0.7	24.4 ± 1.0	7.8 ± 2.3^a^
ANIMALS	score	13.1 ± 1.5	13.7 ± 1.7^e^	11.4 ± 2.2^f^
BNT	score	14.1 ± 0.3	13.9 ± 0.4^e^	12.3 ± 1.4^g^
COWA	score	38.3 ± 3.7	35.0 ± 4.1^d^	28.7 ± 5.7^f^

CTRL: control subjects; aMCI: amnesic mild cognitive impairment subjects; AD: Alzheimer’s disease patients; PMI: *post-mortem* interval; NFTs: neurofibrillary tangles; Aβ: amyloid-beta; MMSE: mini-mental state examination; ANIMALS: animal naming test; BNT: Boston naming test; COWA: controlled oral word association test. Some aMCI and AD patients could not complete some cognitive tests. ^a^*n* = 11; ^b^*n* = 8, ^c^*n* = 5; ^d^*n* = 9; ^e^*n* = 10; ^f^*n* = 7; ^g^*n* = 6.

### Animals

Male and female 3xTg-AD (APPswe, PS1M146V, tauP301L) mice^42^, and control non-transgenic mice of the same genetic background (i.e., C57BL/6-129/SvJ) were produced and maintained at the animal facility of the Research Center of the Centre Hospitalier de l’Université Laval as previously described^43^. Male C57BL/6 J, *Nlgn1* KO mice and wild-type (WT) littermates were used for chronic Aβo_1-42_ injections. C57BL/6 J mice (n = 41) were purchased from Jackson Laboratories and submitted to cannula implantation surgery at 13 weeks (see below). Mice heterozygous for the *Nlgn1* mutation (B6;129-Nlgn1^tm1Bros^/J^44^) were purchased from Jackson Laboratories, backcrossed with C57BL/6 J mice for >10 generations, and bred at the animal facility of the Research Center of the Hôpital du Sacré-Coeur de Montréal. KO and WT mice were implanted with cannulas for intra-hippocampal Aβo injections at 24 ± 10 weeks. Animals were housed individually and maintained in a 12 h light/12 h dark cycle at a temperature of 24 ± 1 °C with food and water available *ad libitum*. The experiment involving 3xTg-AD mice was approved by the Animal Welfare Committee of the Université Laval according to guidelines of the Canadian Council on Animal Care. All other experimental procedures were approved by the *Comité d’éthique de l’expérimentation animale* of the Hôpital du Sacré-Coeur de Montréal (Recherche CIUSSS-NIM) also in accordance with guidelines of the Canadian Council on Animal Care.

### Aβo_1-42_ preparation and Aβ quantification

Preparation of the Aβo_1-42_ and control Aβ_1-42_ scrambled (AβScr) solutions were performed as previously described^12^. Briefly, synthetic Aβ_1-42_ and AβScr (rPeptide, Cat No. A-1163-2 and A-1004-1, respectively) were suspended in hexafluoroisopropanol (HFIP) (Acros Organics, Cat No. 147541000). The HFIP was evaporated with nitrogen gas and Aβ_1-42_ peptides were resuspended in DMSO (Sigma-Aldrich, Cat No. D-4540). The solution was eluted through a HiTrap desalting column (GE Healthcare, Cat No. 17-1408-01) previously equilibrated with a Tris-EDTA solution (50 mM Tris, 1 mM EDTA). Aβ_1-42_ peptides were quantified using the BCA protein assay kit (Thermo Fisher, Cat No. 23225) and stored at −80 °C. The average concentration of the solutions used in this study was 0.28 ± 0.04 μg/μL. The biophysical and biological properties as well as the level of oligomerization of this Aβ preparation have been previously characterized^12,45,46^. The Aβ solution was kept at room temperature for 1 h before injection to induce oligomerization and produce soluble low-molecular-weight Aβo_1-42_ as previously done^12^. Soluble Aβ quantification in post-mortem tissue was performed by a standard three-step serial extraction procedure as described elsewhere for these samples^40^. The Aβ quantification was not available for 4 CTRL, 6 aMCI and 4 AD patients (Table 1).

### Surgical procedure and injections

The cannula implantation surgery was adapted from the procedure previously described^12,47^. Briefly, mice were anesthetized with Ketamine/Xylazine (120/10 mg/kg), installed on a stereotaxic apparatus and maintained under anesthesia using 1% isoflurane. Stereotaxic coordinates of the two cannulas implanted just above the DG were defined as 2.3 mm posterior to the bregma, ±1.5 mm from midline and 1.8 mm below the skull surface. Three screws were inserted into the skull to stabilize cannulas fixed to the skull with dental cement. Mice were given buprenorphine (0.1 mg/kg) immediately after the surgery and the day after to ensure sufficient analgesia.

The injections were conducted as previously described^12,47^. In brief, one week after recovery, freely moving mice were injected with Aβ solutions or an equal volume of control AβScr or vehicle (VH: 50 mm Tris, 1 mm EDTA, pH 7.5) at a constant rate of 0.2 μl/min via cannulas and PE50 tubing (Plastics One) connected to a Hamilton syringe pump system (KD Scientific, KDS310). The tubing was left in place for 5 min after each injection and cannulas were capped after tubing removal to prevent reflux of the injected solution. Injections were performed during the first half of the light phase and were repeated for 2, 4 or 6 consecutive days.

### Protein extraction and Western blot

Proteins extracted from human hippocampi represented the membrane fraction as detailed elsewhere^38^. For mouse experiments, hippocampal tissues were homogenized in cold RIPA buffer 1X (Thermo Fisher, Cat No. 89901) with 0.5% CHAPS (Fisher BioReagents, Cat No. BP571-1) and phosphatase and protease inhibitors (Thermo scientific, Cat No. A32961). Samples were sonicated, gently agitated at 4 °C for 1 h, and centrifuged at 14000 g for 20 min at 4 °C. The supernatant containing total proteins was collected and the protein concentration was quantified using the Pierce BCA protein assay kit. Thirty μg of proteins per sample were separated by electrophoresis using Bolt 4-12% Bis-Tris gels (Thermo Fisher, Cat No. NW04122BOX) and transferred onto a PVDF membrane (Immobilon-P^sq^, Cat No. ISEQ00010). Membranes were incubated 1 h with Odyssey blocking buffer (Type PBS) (LiCor, Cat No. 927-40000) at room temperature and incubated overnight at 4 °C with anti-NLGN1 antibodies (Millipore, Cat No. MABN742, used at 1:1000 for human samples; Synaptic Systems, Cat No. 129 111, used at 1:1000 for all non-human samples), anti-PSD95 antibodies (Millipore, Cat No. 04-1066, 1:1500), or anti-GAPDH antibodies (Cell Signaling Technology, Cat No. 5174, 1:1000) diluted in Odyssey blocking buffer (Type PBS) containing 0.2% Tween 20 (Fisher BioReagents, Cat No. BP337-500). Membranes were then washed and incubated with corresponding secondary antibodies (LiCor, IRDye 800CW, 1:15000; IRDye 680RD, 1:15000; Cell Signaling Technology, HRP linked antibody No. 7076, 1:1000) diluted in Odyssey blocking buffer (Type PBS) containing 0.2% Tween 20 and 0.01% SDS for 1 h at room temperature. Membranes were revealed using the Odyssey CLx (LiCor, Cat No. 9140) with Image Studio 3.1 software. For some blots, chemiluminescence was visualized using an ECL detection kit (Thermo Scientific, Cat No. 1859022). Quantitative densitometric analysis was performed using ImageJ and expressed relative to GAPDH. At least one control sample was loaded on each blot as a calibrator for quantification. Full length blots are presented in Supplementary Information.

### mRNA extraction and qPCR

mRNA extraction, reverse transcription and qPCR procedures were performed as previously described^48–50^. Briefly, hippocampal mRNA was extracted using the RNeasy Lipid Tissue mini kit according to manufacturer’s instructions (Qiagen, Cat No. 74804). Reverse transcription was performed with SuperScript II Reverse transcriptase (Invitrogen, Cat No. 18064014) following the manufacturer’s instructions including 10 min at 25 °C and 60 min at 42 °C. QPCR was performed using 384-well plates with a mix of RNAase free water, TaqMan Fast Advanced Master Mix (Applied Biosystems, Cat No. 4444557), and primers and probes for each gene (Table 2). Given that NLGN1 has isoforms with different properties^51^, five different probe sets were used to quantify *Nlgn1* expression as previously performed^48–50^. *Nlgn1A* corresponds to the sequence containing insert A and *Nlgn1B* with insert B, while *Nlgn1NA* is a sequence without insert A and *Nlgn1NB* without insert B. *N1gn1C* corresponds to a common sequence targeting transcripts with only insert A, only insert B, both inserts and no insert. The expression of four additional genes coding for related synaptic proteins have also been quantified (*Nlgn2*, *Nrxn1*, *Nrxn2*, *Nrxn3*). Three endogenous control genes (*GusB*, *Rps9* and *Tbp*) were quantified to normalize expression level of *Nlgn1* variants and of related mRNA. Gene expression was measured using a Viia 7 thermal cycler (Thermo Fisher) with the following cycling conditions: 2 min at 50 °C, 20 sec at 95 °C, 40 times 1 sec at 95 °C and 20 sec at 60 °C. The analysis was performed with Expression Suite (Life Technologies), which calculated gene expression relative to endogenous controls and to one sample of the control group (modified delta delta Ct).

**Table 2:** List of probes and primers used for qPCR.

Gene	Type	Manufacturer	Sequence (5′ to 3′) or Taqman Gene Expression Assay number
*GusB*	Forward	Operon	ACGGGATTGTGGTCATCGA
	Reverse	Operon	TGACTCGTTGCCAAAACTCTGA
	Probe	Operon	[6-FAM]-AGTGTCCCGGTGTGGGCATTGTG-[TAMRA]
*Nlgn1A*	Forward	Microsynth	ACGGTGCTGAAGATGAAG
	Reverse	Microsynth	CAGTACCTTCCATGTAAGAG
	Probe	Eurogentec	[6-FAM]-TCCCAAACCAGTGATGGTGTACATCCA-[BHQ1]
*Nlgn1NA*	Forward	Microsynth	GGATGTGGTTTCATCATAC
	Reverse	Microsynth	TGTCCCGAATATCATCTTC
	Probe	Eurogentec	[6-FAM]-TCCAAGACCAGAGTGAAGACTGTC-[BHQ1]
*Nlgn1B*	Forward	Microsynth	GGTAACCGTTGGAGCAATTCA
	Reverse	Microsynth	CCAGCTGGAAAGGGCTGTT
	Probe	Eurogentec	[6-FAM]-CCAAAGGACTTTTTCAACGAGCAATAGCTCA-[BHQ1]
*Nlgn1NB*	Forward	Microsynth	CACTGTGTTTGGATCAGG
	Reverse	Microsynth	AAAAGTCCTTCAGAATAATGGG
	Probe	Eurogentec	[6-FAM]-GGTTCATGTGTCAACCTGCTGACT-[BHQ1]
*Nlgn1C*	Forward	Microsynth	TCCTTGCAATTTCTCCAAGA
	Reverse	Microsynth	TTGGGTTTGGTATGGATGAA
	Probe	Eurogentec	[6-FAM]-TGGTGACCCAAATCAACCAGTTCC-[BHQ1]
*Nlgn2*	Probe set	Thermo Sci.	Mm01703404_m1
*Nrxn1*	Probe set	Thermo Sci.	Mm00660298_m1
*Nrxn2*	Probe set	Thermo Sci.	Mm01236851_m1
*Nrxn3*	Probe set	Thermo Sci.	Mm04279482_m1
*Rps9*	Forward	Operon	GACCAGGAGCTAAAGTTGATTGGA
	Reverse	Operon	TCTTGGCCAGGGTAAACTTGA
	Probe	Operon	[6-FAM]-AAACCTCACGTTTGTTCCGGAGTCCATECT-[TAMRA]
*Tbp*	Forward	Operon	TTGACCTAAAGACCATTGCACTTC
	Reverse	Operon	TTCTCATGATGACTGCAGCAAA
	Probe	Operon	[6-FAM]-TGCAAGAAATGCTGAATATAATCCCAAGCG-[TAMRA]

*BHQ1: Black Hole Quencher-1 GusB*: beta-glucuronidase B; *Nlgn1A*: neuroligin-1 with insert in splice site A; *Nlgn1NA*: neuroligin-1 without insert in splice site A; *Nlgn1B*: neuroligin-1 with insert in splice site B; *Nlgn1NB*: neuroligin-1 without insert in splice site B; *Nlgn1C*: neuroligin-1 common to all transcript variants; *Nlgn2*: neuroligin-2; *Nrxn1*: neurexin-1; *Nrxn2*: neurexin-2; *Nrxn3*: neurexin-3; *Rps9*: ribosomal protein S9; *Tbp*: TATA-box binding protein; FAM: 6-carboxyfluorescein; TAMRA: tetramethylrhodamine.

### Primary hippocampal culture and cell viability

The primary neuronal culture procedure was carried out as described previously^12^. Briefly, pregnant Long-Evans female rats were ordered from Charles River Laboratories and sacrificed at embryonic day 18. Hippocampi were dissected, transferred to a tube containing HBSS (Sigma-Aldrich, Cat No. H1641), HEPES (Gibco, Cat No. 15630-080), penicillin/streptomycin/amphotericin (Gibco, Cat No. 15240-062) and 3 mM sodium pyruvate (Gibco, Cat No. 25080-094) at 37 °C, and were treated with 0.25% trypsin (Life Technologies, Cat No. 15090-046), agitated at 900 rpm for 15 min at 37 °C, and dissociated by trituration. Cells were platted in 12-well plates (Costar, Cat No. 3513) coated with poly-D lysine hydrobromide (Sigma-Aldrich, Cat No. P6407- 5MG) and containing minimum essential medium (Sigma-Aldrich, Cat No. M0275) supplemented with 10% horse serum (Sigma-Aldrich, Cat No. H1138), 3 mM sodium pyruvate, 25 mM D-Glucose (Sigma, Cat BP-250), 2 mM GlutaMax (Sigma, Cat No. RNBD9302), and 1% penicillin/streptomycin/amphotericin. Plates were incubated at 37 °C with 5% CO_2_ overnight, and the initial culture medium was replaced the day after with Neurobasal medium (Gibco, Cat No. 21103-049) supplemented with 2% B27 (Gibco, Cat No. 17504-044), 2 mM GlutaMax, and 0.5% penicillin/streptomycin/amphotericin. Primary hippocampal cells were cultured for 7 days before starting treatments. For Western blot analysis, cells were washed with cold 10 mM PBS, scratched off the wells with 140 uL of cold RIPA 1X buffer containing CHAPS and phosphatase and protease inhibitors, and rapidly stored at −80 °C. Protein extraction and Western blotting were performed as described above.

Cell viability was measured using the CellTiter 96 AQ_ueous_ One Solution Cell Proliferation Assay (Promega, Cat No. G358B) according to the manufacturer’s protocol. Briefly, the CellTiter 96 AQ_ueous_ One Solution reagent was added to the Neurobasal medium containing the cells (v/v ratio of 1:5) and after 1.5 h of incubation at 37 °C the absorbance was recorded at 490 nm. The tested conditions were normalized to values obtained with water-treated wells.

### Spatial object recognition (SOR) test

The SOR task was conducted 24 hours after the last hippocampal injection (Supplementary Fig. 1) in *Nlgn1* KO mice and wild-type (WT) littermates similar to previously described^52^. It was done in an opaque plastic arena (45 × 45 cm) with the floor covered with litter that was mixed after each trial to eliminate olfactory cues. The four walls contained different visual cues and two different objects were placed in adjacent corners of the arena. The walls and objects were cleaned with 70% ethanol between each mouse. During the first day, mice explored the arena and objects for 5 min. Twenty-four hours later, one of the two objects was moved to the opposite corner of the arena and mice were reintroduced in the arena for 5 min. The number of interactions with each object, defined as a touch of the snout or sniffing of the object by the mouse, was counted by an experimenter blind to the genotype and treatment. The discrimination index (DI) was calculated for the number of interactions according to the following formula: (moved object - fixed object)/(moved object + fixed object).

### Y-maze test

A two-trial Y-maze task was performed 48 hours after the SOR test (Supplementary Fig. 1) as previously described^12^. The three arms of the Y maze were 39 cm long, 15 cm wide and 40.5 cm high. The floor of the maze was covered with litter and different visual cues were placed at the top of the end of each arm. During the first trial, mice were placed at the end of one arm and were allowed to explore the two arms of the maze freely during 5 min without access to the third arm. The blocked arm was alternated between each mouse. Mice were returned to their home cage for 5 min and then reintroduced into the same starting arm of the Y-maze to explore all three accessible arms for 2 min. Movements of the mouse were analyzed using the Smart acquisition software (Harvard Apparatus) to extract time spent in the new arm and the average time spent in the other two arms. The walls were cleaned with 70% ethanol and the litter was mixed between each mouse to avoid olfactory cues.

### Morris water maze (MWM) test

The MWM task was conducted 48 hours after the Y-maze test (Supplementary Fig. 1) as previously described^53^. Mice were trained in a 120-cm diameter pool filled with water rendered opaque by adding non-toxic white paint. The temperature of the water was kept at 24 ± 1 °C during testing. During the first 4 days, mice had to learn to find a submerged platform in the pool using visuo-spatial cues installed around the pool. Mice had 3 trials per day with 30 min between each trial. If the mouse failed to reach the platform within 60 sec, it was guided to the platform and had to remain on the platform for 10 sec before being removed from the pool. The time and distance to reach the platform were extracted by the Smart software (Harvard Apparatus). Starting location of the mice was different for each trial. During the probe trial on the fifth day, the platform was removed and mice had 60 sec to explore the pool. The time spent in the target quadrant that previously contained the platform and in the other quadrants, as well as the number of times the mouse crossed the area around the platform (2x the diameter of the platform) were calculated with the Smart software. A final cued task of 3 trials was also performed to verify visual acuity and motivation to escape from water. During this test, mice had a maximum of 30 sec to reach a visible platform that was moved in a different quadrant between each trial. Data were acquired in real time with the Smart software.

### Immunofluorescence (IF) staining

Neurons were marked with the NeuN antibody for neuronal counting purpose^54^, as described below. Staining also served to validate the adequate positioning of cannulas. Brains were cut with a HM525NX cryostat (Thermo Scientific, Cat No. 956640) at −20 °C and 10 μm coronal slices were mounted on SuperFrost Plus slides (Fisher Scientific, Cat No. 12-550-15). Hippocampal sections were performed at ±100 μm from the middle of the injection site. Brain slices were mounted in series on 6 slides, with 4 slices per slide. Slides were fixed for 15 min in 10% formalin (Sigma-Aldrich, Cat No. HT501128) and washed with PBS (Cell Signaling Technology, Cat No. 9808S). Sections were immersed in a blocking solution (1X PBS, 5% goat serum (Cell Signaling Technology, Cat No. 5425), 0.3% Triton X-100 (Fisher BioReagents, Cat No. BP151-500) for 60 min at room temperature and incubated overnight at 4 °C with anti-NeuN antibodies (Cell Signaling, Cat No. 24307; 1:1000) diluted in PBS 1X, 1% BSA (Fisher BioReagents, Cat No. BP1600-100) and 0.3% Triton X-100. The slides were washed, and incubated with a secondary antibody (Alexa fluor 488, Cell Signaling Technology, Cat. No. 4340, 1:1000) for 90 min at room temperature and protected from light. Slides were then washed, dried and one drop of Prolong Gold Antifade DAPI (Cell Signaling Technology, Cat No. 8961S) was applied on each section before being covered with a coverslip (Thermo Scientific, Cat No. 102455).

### Microscopy

A Zeiss AxioImager 2 fluorescence microscope, a monochrome camera, and the StereoInvestigator software (MBF Biosciences) were used to acquire IF images. Wavelength filters of 488 nm and 350 nm were used to excite the fluorochromes of the secondary antibody and of DAPI. Neuronal counting was performed using the Neurolucida Software (MBF Biosciences). The 20X objective was used to precisely identify the depth of the injection site. Two mice were excluded from neuronal count and memory assessments because the cannula was positioned outside the target region. Using the 40X objective, a 500 μm wide area was defined in the DG under the injection site as well as in CA2-CA3 distant to the injection site. Since there was little or no cell overlay using 10 μm sections and that NeuN labeling was well defined, the function Detect Cells of the Neurolucida program could discriminate between individual cells. This function made it possible to make a reliable cell count in the area under the injection site and the distant area, which has been validated by manual counting by a blind experimenter. Three to four sections per slide were counted; the average of these sections was calculated and analyzed according to genotype and treatment.

### Statistical analyses

Statistical analyses of multiple samples were performed using appropriate analyses of variance (ANOVAs). One-way ANOVA was used for human sample analysis; two-way ANOVAs were used to analyze results of 3xTg-AD mice, cell culture, chronic injections, IF, and most variables of the SOR and MWM tests; three-way ANOVA was used for the MWM training phase. Significant effects were decomposed, when appropriate, using planned comparisons. Student t tests were used for some analyses of the SOR and the Y-maze. Pearson’s correlations were performed to quantify the association between soluble Aβ, MMSE and NLGN1 level. The sizes of the different samples were determined on the basis of previous studies in the field. Statistical analyses were performed using GraphPad Prism 6.0 and/or JMP 14. Significance threshold was set at 0.05 and the levels of statistical significance are identified with *p < 0.05, **p < 0.01, ***p < 0.001. Data are reported as mean ± SEM. The number of samples used for each experiment is reported in figure legends.

## Results

### Lower NLGN1 in AD patients

**Figure 1:**
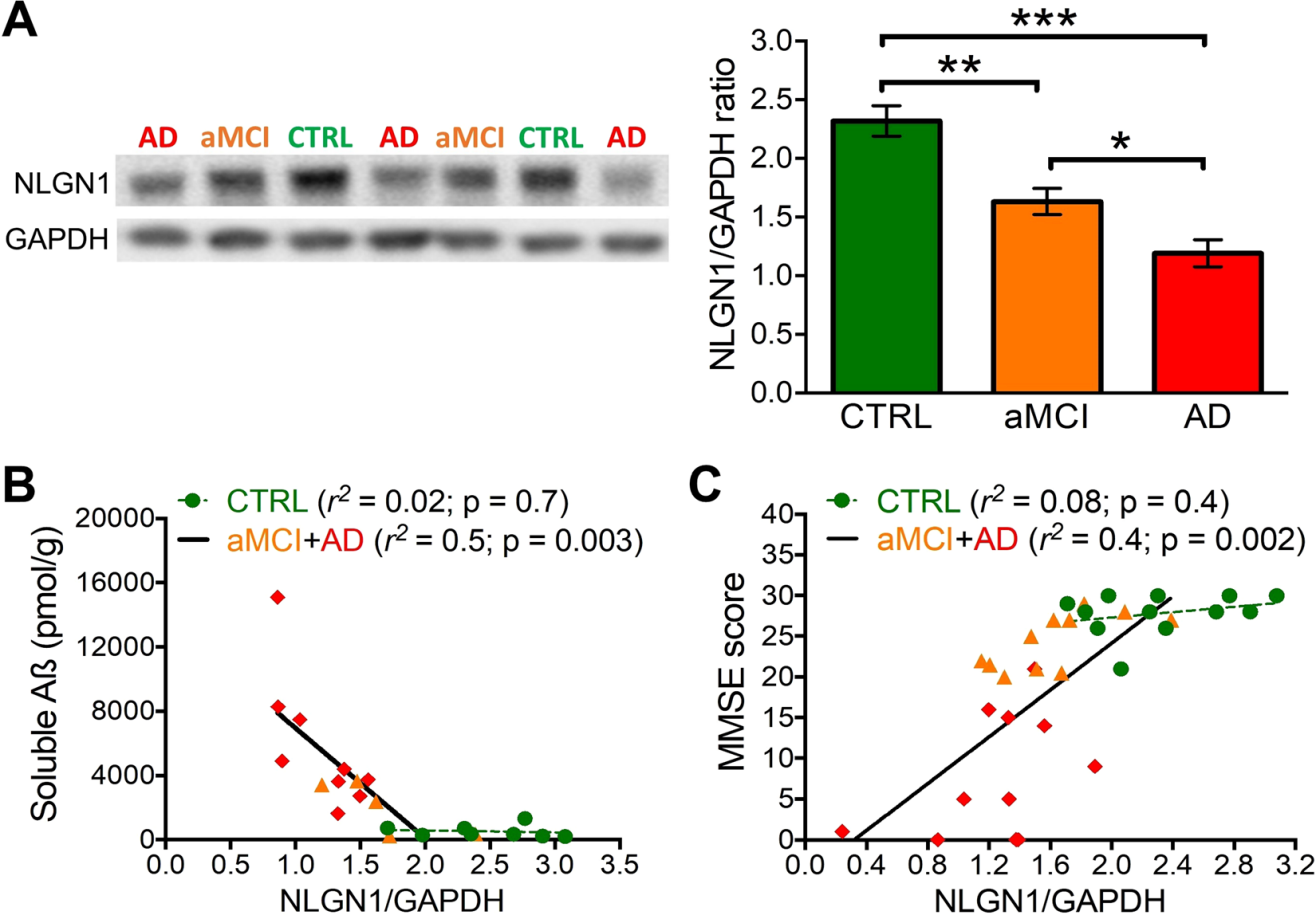
NLGN1 hippocampal level is decreased in aMCI individuals and AD patients. (**A**) NLGN1 level was measured in aMCI individuals (*n* = 11), AD patients (*n* = 13), and control aged non-demented subjects (CTRL, *n* = 12) by Western blot (left side) and quantified (right side) using GAPDH protein as a loading control. Significant differences are represented between indicated groups (one-way ANOVA F_2,33_ = 23.3; p < 0.0001; stars indicating planned comparisons). Full length blots are shown in Supplementary Fig. 2. (**B**) Pearson correlation between soluble Aβ and NLGN1 level showed a significant negative correlation in aMCI individuals (*n* = 5) and AD patients (*n* = 9) but not in CTRL (*n* = 8). (**C**) Pearson correlation between MMSE score and NLGN1 level showed a significant positive correlation in aMCI individuals (*n* = 11) and AD patients (*n* = 11) but not in CTRL (*n* = 12).

We first assessed whether the hippocampal level of NLGN1 is decreased in AD patients and in individuals with aMCI, a prodromal stage of AD^55,56^. We found that aMCI individuals have lower levels of NLGN1 in the hippocampus compared to age-matched CTRL without cognitive impairment (p < 0.001; Fig. 1A, Supplementary Fig. 2A). Also, NLGN1 level was found to be significantly lower in AD patients when compared to both aMCI individuals (p < 0.05) and CTRL (p < 0.001). These results suggest that there is a decline in human hippocampal NLGN1 level that is matching disease progression. In addition, when expressed relative to the synaptic marker PSD95, NLGN1 was significantly decreased in both aMCI and AD in comparison to CTRL (p < 0.05; Supplementary Fig. 2B,C), which indicates that the NLGN1 decrease is larger than that of PSD95 previously reported to occur early during the neurodegenerative process^53^. Moreover, we found that, in aMCI individuals and AD patients, lower hippocampal level of NLGN1 significantly correlates with higher hippocampal level of soluble Aβ (p < 0.01; Fig. 1B) as well as with poorer cognitive state assessed with the MMSE (p < 0.01; Fig. 1C).

### Lower NLGN1 level in young 3xTg-AD female mice

**Figure 2:**
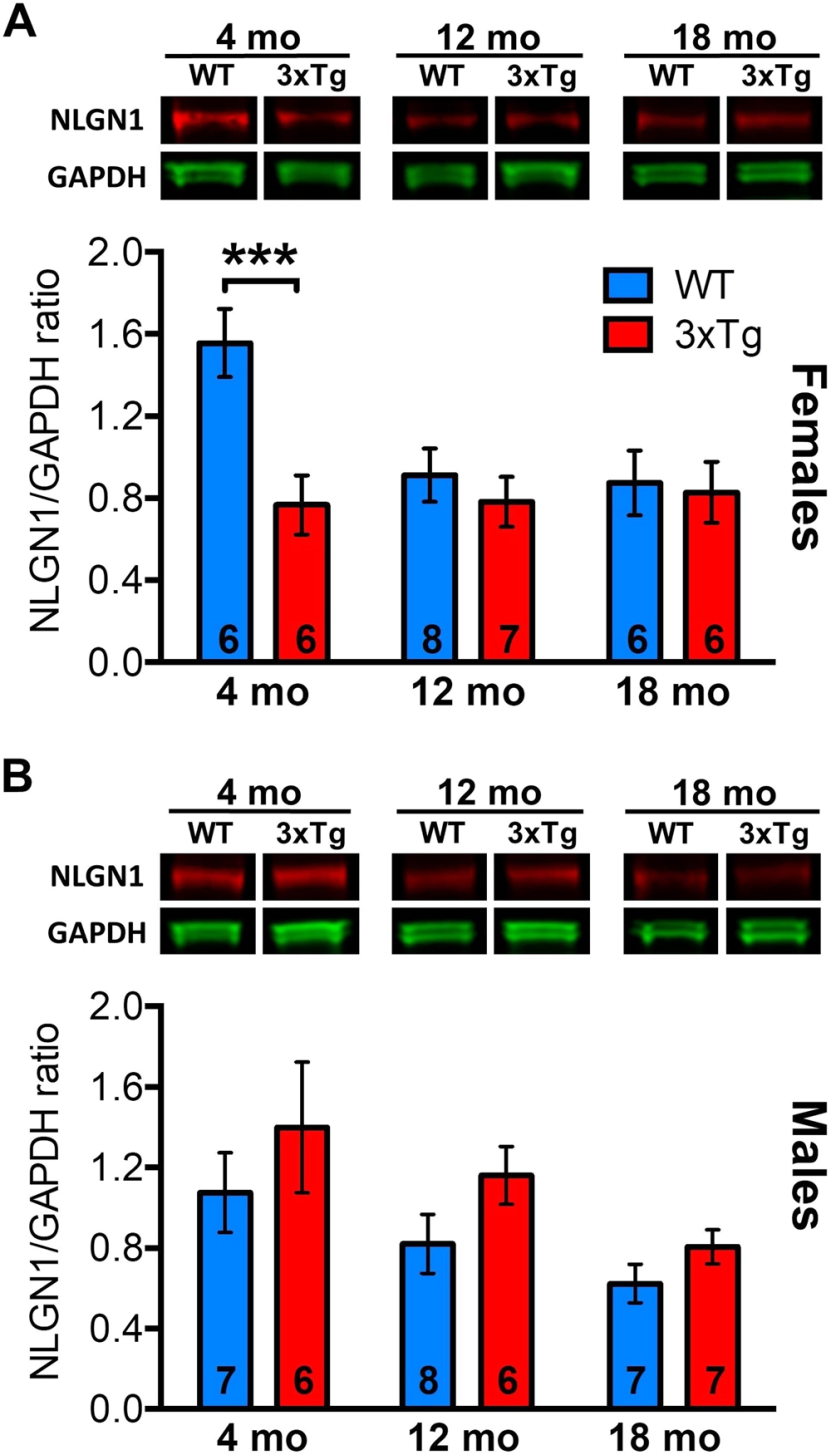
Age-dependent changes in NLGN1 protein in 3xTg-AD mice. (**A**) Hippocampal NLGN1 protein level measured by Western blot (top) in female 3xTg-AD and control mice at 4, 12 and 18 months, and analyzed using GAPDH protein as a control (bottom). A significant Genotype-by-Age interaction was found (two-way ANOVA F_2,33_ = 3.8, p = 0.03) showing that NLGN1 protein level differed between 3xTg-AD and control mice only at 4 months (stars indicating planned comparison). Full length blots are shown in Supplementary Fig. 3 (also in B). (**B**) Hippocampal NLGN1 protein level measured by Western blot in male 3xTg-AD and control mice at 4, 12 and 18 months (top), and analyzed using GAPDH as a control (bottom). No significant effect of Genotype was found (two-way ANOVA Genotype main effect: F_1,35_ = 3.9, p = 0.06; Genotype-by-Age interaction: F_2,35_ = 0.12, p = 0.9), but a significant effect of age was observed (Age main effect: F_2,35_ = 4.4 p = 0.02). The number of mice is indicated on each bar.

To further evaluate the fate of NLGN1 in the course of neurodegeneration, we measured NLGN1 hippocampal levels in 3xTg-AD male and female mice at three different ages (4, 12 and 18 months). This commonly used AD model shows beta-amyloid plaques and neurofibrillary tangles with intracellular Aβ accumulation in the hippocampus at 4 months that correlates with cognitive deficits^42,57^. Female 3xTg-AD mice showed considerably lower NLGN1 level at 4 months in comparison to control female (p < 0.001; Fig. 2A, Supplementary Fig. 3). NLGN1 level did not significantly differ between female 3xTg-AD and control mice at 12 and 18 months. In male mice, NLGN1 hippocampal level did not significantly differ between 3xTg-AD and controls at all ages studied (Fig. 2B, Supplementary Fig. 3). Interestingly, NLGN1 level decreased with age in males, with a significant difference specifically observed between 4 and 18 months (p = 0.01). In summary, these data indicate that NLGN1 is rapidly decreased in 3xTg-AD mice, and that this decrease is specific to female mice.

### NLGN1 level is decreased by Aβo_1-42_ exposure *in vitro*

**Figure 3:**
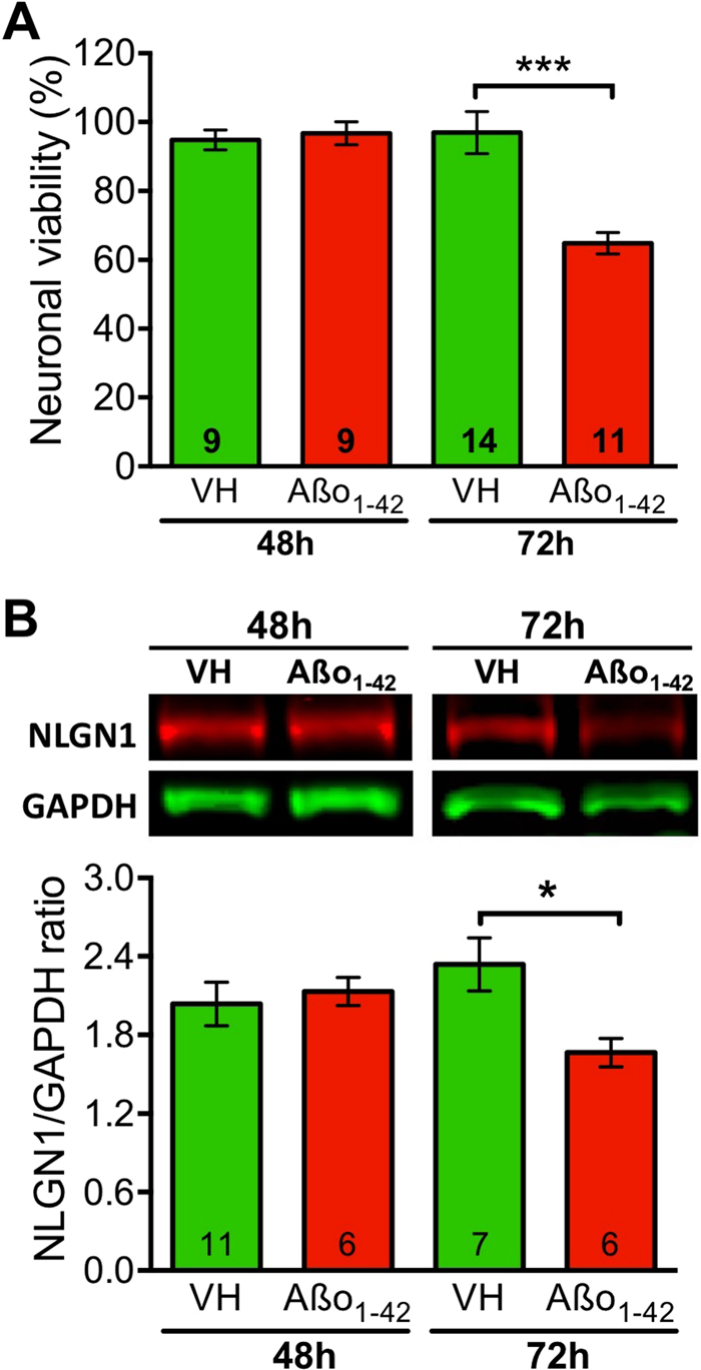
Aβo_1-42_ exposure impacts cell viability and NLGN1 in primary neuronal culture. (**A**) Neuronal viability of hippocampal neurons exposed to 2 µM of Aβo_1-42_ or vehicle (VH) for 48 and 72 hours. A significant Treatment-by-Time interaction was found (two-way ANOVA F_1,39_ = 12.6, p = 0.001), with difference between Aβo_1-42_ and VH restricted to 72 hours of treatment (stars indicating planned comparison, also in B). (**B**) NLGN1 protein level in primary culture of hippocampal neurons was assessed by Western blot (top) and expressed relative to the control protein GAPDH (bottom) after exposure to 2 µM of Aβo_1-42_ or VH for 48 and 72 hours. A significant Treatment-by-Time interaction was also found (two-way ANOVA F_1,22_ = 5.43, p = 0.03), with difference between Aβo_1-42_ and VH also restricted to 72 hours of treatment. Full length blots are shown in Supplementary Fig. 4. The number of replicate is indicated on each bar.

Given our observation that NLGN1 level significantly correlates with soluble Aβ in the human hippocampus (Fig. 1B), we next verified whether Aβo_1-42_ could directly impact NLGN1 level *in vitro*, and whether this is linked to neuronal survival. NLGN1 protein level and cell viability were measured in primary culture of hippocampal neurons treated with 2 μM of Aβo_1-42_ for 48 or 72 hours. As expected from our previous study^12^, Aβo_1-42_ significantly decreased neuronal viability by about 40% after 72 hours of treatment (p < 0.001; Fig. 3A). No change in viability was observed after 48 hours of treatment. NLGN1 protein level was also decreased after 72 hours of Aβo_1-42_ exposure (p < 0.02) and unchanged after 48 hours (Fig. 3B, Supplementary Fig. 4). These data indicate that Aβo_1-42_ exposure is detrimental to NLGN1 protein level in hippocampal neurons, which follows a time course similar to neuronal viability.

### *Nlgn1* mRNA is affected by hippocampal Aβo_1-42_ injections

**Figure 4:**
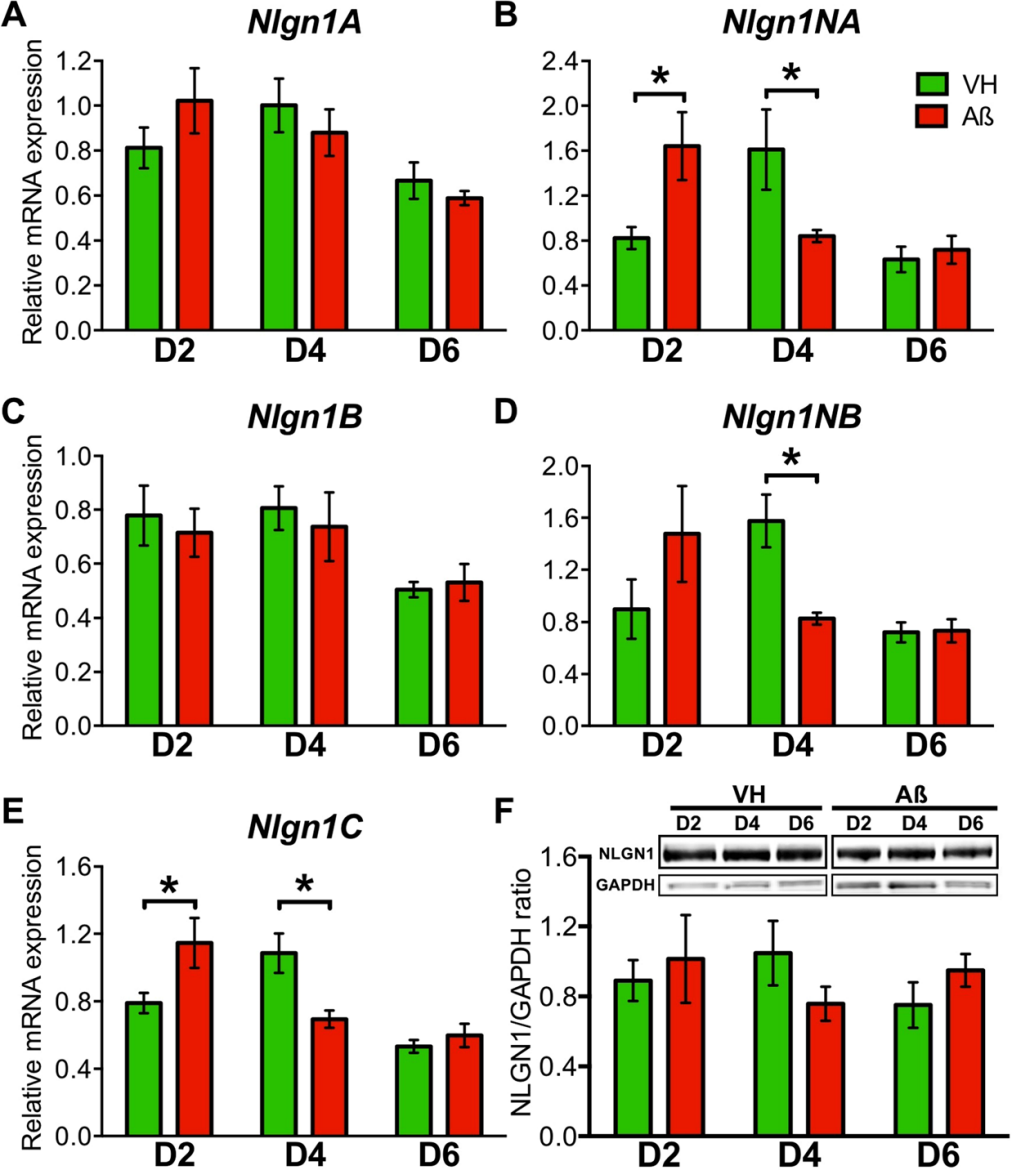
Chronic hippocampal injections of Aβo_1-42_ change *Nlgn1* mRNA expression. *Nlgn1* mRNA and protein expression was measured after 2, 4 or 6 days of hippocampal injections of Aβo_1-42_ or vehicle (VH). (**A**) *Nlgn1A* expression showed no change with treatment throughout the 6 days (two-way ANOVA Treatment main effect: F_1,30_ = 0.0009, p = 0.98; Treatment-by-Day interaction: F_2,30_ = 1.6, p = 0.2). (**B**) *Nlgn1NA* expression showed a significant Treatment-by-Day interaction (two-way ANOVA F_2,29_ = 4.77, p = 0.02) with significant difference between Aβo_1-42_ and VH after 2 and 4 days (stars indicating planned comparison, also in D and E). (**C**) *Nlgn1B* expression showed no change with treatment throughout the 6 days (two-way ANOVA Treatment main effect: F_1,30_ = 0.2, p = 0.6; Treatment-by-Day interaction: F_2,30_ = 0.2, p = 0.8). (**D**) *Nlgn1NB* expression showed a significant Treatment-by-Day interaction (two-way ANOVA F_2,28_ = 3.4, p < 0.05) with significant difference between Aβo_1-42_ and VH after 4 days. (**E**) *Nlgn1C* expression showed a significant Treatment-by-Day interaction (two-way ANOVA F_2,28_ = 8.0, p = 0.002) with significant difference between Aβo_1-42_ and VH after 2 and 4 days. (**F**) Hippocampal NLGN1 protein level and loading control GAPDH measured by Western blot after chronic injections (top) and the quantification (bottom). No change was found with treatment throughout the 6 days (two-way ANOVA Treatment main effect: F_1,28_ = 0.001, p = 0.9; Treatment-by-Day interaction: F_2,28_ = 0.22, p = 0.8). Full length blots are shown in Supplementary Fig. 6. The number of mice is 5–6 per group.

We next aimed at identifying the specific impact of Aβo_1-42_ on NLGN1 *in vivo* using chronic (2, 4 or 6 days) hippocampal injections in WT mice. In addition, we assessed whether Aβo_1-42_ affect *Nlgn1* in a transcript variant-dependent manner, which can be done by measuring mRNA expression using different probe sets targeting the presence or absence of the two NLGN1 inserts^48–50^. Two days of Aβo_1-42_ injections significantly increased the hippocampal expression of amplicons *Nlgn1NA* (p < 0.04) and *Nlgn1C* (p < 0.02) in comparison to control injections (Fig. 4B,E); whereas 4 days of injections significantly decreased the expression of amplicons *Nlgn1NA* (p < 0.04), *Nlgn1NB* (p < 0.05) and *Nlgn1C* (p < 0.01) (Fig. 4B,D,E). The *Nlgn1A* and *Nlgn1B* amplicons were not significantly changed by Aβo_1-42_ injections (Fig. 4A,C). Also, hippocampal Aβo_1-42_ injections did not significantly affect the gene expression of the related synaptic proteins NLGN2, NRXN1, NRXN2 and NRXN3 (Supplementary Fig. 5). NLGN1 protein level, which was measured in the hippocampus of the other hemisphere, showed a tendency to be decreased by 4 days of Aβo_1-42_ injections similar to specific *Nlgn1* amplicons (Fig. 4F, Supplementary Fig. 6), but the change did not reach statistical significance (p < 0.2). These results suggest a transcript variant-specific impact of Aβo_1-42_ on the expression of *Nlgn1*, and that this impact shows a bi-phasic time course with an initial increase followed by a decrease.

### NLGN1 deletion worsens spatial learning deficits after treatment with Aβo_1-42_

We next tested the functional role of NLGN1 specifically in Aβo-induced *in vivo* damage. We first investigated the implication of NLGN1 specifically in Aβo-driven memory impairment using assessments of spatial and working memory, selected for their dependency on the hippocampus^58,59^. This was done using *Nlgn1* KO mice and WT littermates submitted solely to 4 days of injections of Aβo_1-42_ or AβScr (Supplementary Fig. 1). The condition with 4 injection days was selected given that it specifically decreased expression of precise *Nlgn1* transcripts (Fig. 4) and to obtain an exposure to Aβo_1-42_ intermediate in comparisons to 6 days that was shown to induce substantial neuronal loss and memory deficits^12^.

**Figure 5:**
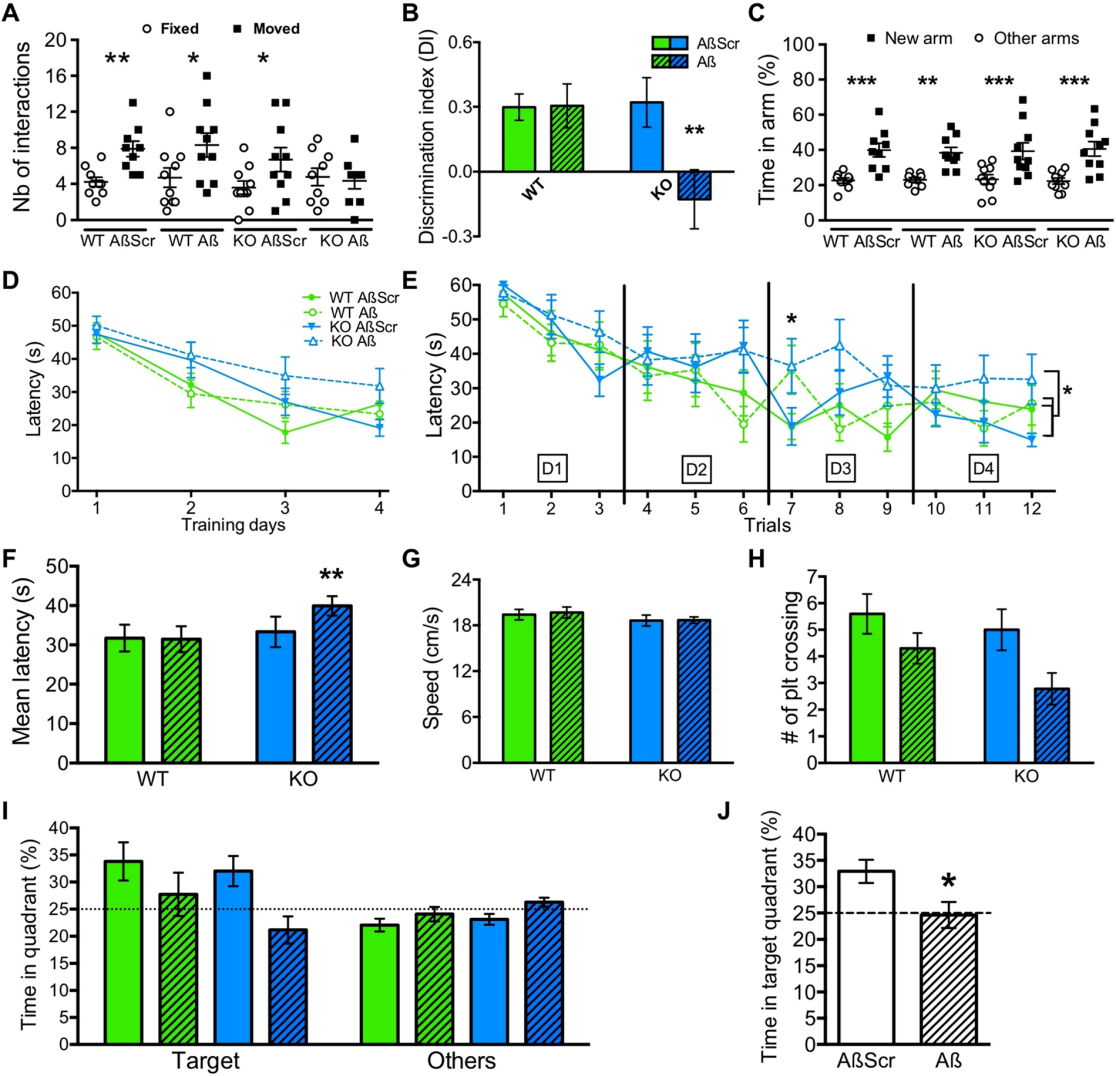
NLGN1 absence impairs spatial memory after Aβo_1-42_ exposure. (**A**) Number of interactions with the fixed and moved objects in the SOR task for WT and *Nlgn1* KO mice treated with Aβo_1-42_ or AβScr. Stars indicate significant differences between moved and fixed objects (t ≥ 2.6). (**B**) A Genotype-by-Treatment interaction was found for the discrimination index (DI) in the SOR task (two-way ANOVA F_1,34_ = 4.5, p = 0.04; stars indicate planned comparisons, also in E, F and J). (**C**) Percent time in the new and other arms in the Y-maze task for the four groups. Stars indicate significant differences between new and other arms (t ≥ 2.9). (**D**) Latency to the platform during the MWM training days showed significant effects of Genotype (three-way ANOVA F_1,159_ = 5.4, p = 0.02) and Day (F_3,151_ = 25,2, p < 0.0001) but no significant Genotype-by-Treatment-by-Day interaction (F_3,159_ = 0.4, p = 0.7). (**E**) Latency to the platform during the MWM training trials showed significant Treatment-by-Trial (three-way ANOVA F_3,151_ = 2.9, p = 0.03) and Genotype-by-Treatment (F_1,155_ = 3.9, p < 0.05) interactions. (**F**) Graph of panel E Genotype-by-Treatment interaction showing the mean latency of all 12 trials. (**G**) Swimming speed during the MWM probe phase (two-way ANOVA Treatment F_1,35_ = 0.06, p = 0.8; Genotype F_1,35_ = 1.8 p = 0.2; interaction F_1,35_ = 0.03, p = 0.9). (**H**) Number of platform crossing during the MWM probe phase showed a significant Treatment effect (two-way ANOVA F_1,39_ = 6.2 p = 0.02). (**I**) Percent time in the target and other quadrants during the MWM probe test showed a significant Treatment-by-Quadrant interaction (three-way ANOVA F_1,77_ = 8.6, p = 0.0045). (**J**) Percent time in the target quadrant showed for Aβo_1-42_ and AβScr injected animals. The number of mice is 9-10 per group.

First, SOR was differentially affected by Aβo_1-42_ in *Nlgn1* KO and WT mice. Indeed, the number of interactions with the moved object was significantly higher than the number of interactions with the fixed objects for WT mice injected with AβScr (p = 0.004), WT mice injected with Aβo_1-42_ (p = 0.01) and KO mice injected with AβScr (p = 0.02), indicating memory for spatial location (Fig. 5A). However, *Nlgn1* KO mice injected with Aβo_1-42_ did not differ in the number of interactions between the moved and fixed objects (p = 0.6), suggesting impaired spatial memory (Fig. 5A). This is also reflected in the DI, which was significantly lower in KO mice injected with Aβo_1-42_ than in all other groups (p = 0.002 vs. *Nlgn1* KO mice with control injections; p = 0.001 vs. WT mice with control injections; p = 0.002 vs. WT mice with Aβo_1-42_ injections; Fig. 5B). These results indicate that only animals lacking NLGN1 express SOR memory deficits with intermediate exposure to Aβo_1-42_. In contrast, working memory in the Y-maze did not significantly differ between *Nlgn1* KO and WT mice both under control and Aβo_1-42_ injections (Fig. 5C). All four groups indeed spent more time exploring the new arm in comparison to the other arms (p ≤ 0.001), which indicates preservation of working memory independent of the presence of NLGN1. The total distance traveled during the Y-maze test was equivalent between groups, whereas KO mice showed a small, but significant, decreased in the distance traveled during the SOR test (Supplementary Fig. 7).

Spatial memory was further tested in the MWM. As expected for the learning phase, the latency to reach the platform progressively decreased over training days (Fig. 5D). This decrease did not significantly differ between *Nlgn1* KO and WT mice with Aβo_1-42_ and control injections, but the latency was globally higher in KO mice in comparison to WT mice (p = 0.02). When all trials of the 4 training days are considered separately (Fig. 5E), Aβo_1-42_ injected WT and KO mice showed a longer latency to reach the platform compared to AβScr injected WT and KO mice on the first trial of day 3 (p < 0.01). The first trial of each day was proposed to reflect memory retention^57^, for which deficits could be induced by Aβo_1-42_ independent of the genotype. Most importantly, *Nlgn1* KO mice injected with Aβo_1-42_ showed a significant overall higher latency to the platform than all other groups (p < 0.01 vs. *Nlgn1* KO mice with control injections; p < 0.01 vs. WT mice with control injections; p < 0.001 vs. WT mice with Aβo_1-42_ injections), which is also illustrated in Fig. 5F. These findings are reminiscent of observations in the SOR task and further support a greater impact of Aβo_1-42_ on spatial learning in the absence of NLGN1.

In the MWM probe phase, the platform is removed, and the number of times mice are crossing the zone where it was and the time spent in the quadrant where it was located (target quadrant) are indicative of spatial memory. Beforehand, we verified that the swimming speed did not significantly differ between WT and KO mice with Aβo_1-42_ and control injections (Fig. 5G). This confirmed equivalent locomotion across genotypes and treatments, and excluded this potential confounding in the evaluation of memory. We found that Aβo_1-42_ significantly reduced the number of platform crossing in the probe test independent of the genotype (p = 0.02; Fig. 5H). Similarly, Aβo_1-42_ injected animals spent a lower percentage of time in the target quadrant compared to AβScr injected animals (p < 0.01; Fig. 5I,J). Indeed, Aβo_1-42_ injected WT and KO mice were not different from the 25% time spent in quadrant, which represents chance, while AβScr injected mice clearly spent more than 25% time in the target quadrant (Fig. 5J). Taken together, results from the MWM suggest that the absence of NLGN1 worsens the impact of Aβo_1-42_ on learning, but not on the final acquired memory.

### NLGN1 deletion reveals neuronal loss after treatment with Aβo_1-42_

**Figure 6:**
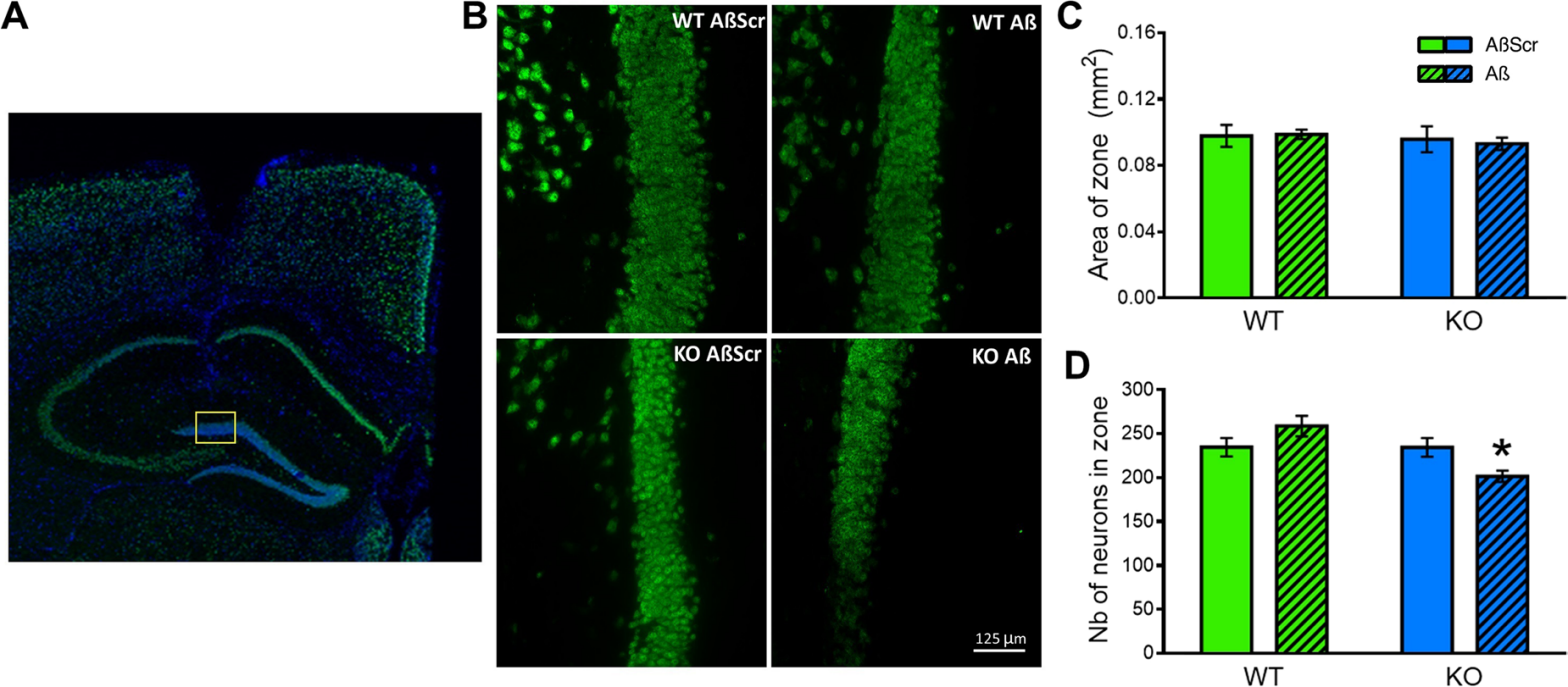
NLGN1 absence induces neuronal loss after Aβo_1-42_ exposure. (A) Representative IF images of NeuN staining (green) in the full hippocampus. The approximate location of the counting area is indicated by the yellow rectangle. Magnification 2.5X. (**B**) Representative IF images of NeuN staining (green) in the DG, where neuron counting was conducted. One image per group is showing approximately the same position within the DG. Magnification 40X. (**C**) Quantification of the area covered by the counting zone, which did not significantly differ between groups (two-way ANOVA Genotype main effect: F_1,20_ = 0.6, p = 0.4; Treatment main effect: F_1,20_ = 0.1 p = 0.8; Genotype-by-Treatment interaction: F_1,20_ = 0.2, p = 0.7). (**D**) Number of NeuN-marked cells counted in the DG target area showed a significant Genotype-by-Treatment interaction (two-way ANOVA F_1,20_ = 8.1, p = 0.01), with the KO Aβ group showing a significant difference in the number of immunoreactive cells in comparison to all other groups (star indicates planned comparisons). The number of mice is 6 per group.

Lastly, we asked whether the absence of NLGN1 impacts neuronal loss in the DG caused by chronic hippocampal Aβo_1-42_ injections. This was assessed using the same mice as above tested for spatial and working memory. Neuronal loss was measured in the DG in an area located under the injection site (Fig. 6). The size of the counting area did not differ between groups (Fig. 6C). However, DG neuronal count was significantly decreased in *Nlgn1* KO mice submitted to Aβo_1-42_ injections in comparison to all other groups (p = 0.02 vs. *Nlgn1* KO mice with control injections; p = 0.02 vs. WT mice with control injections; p = 0.002 vs. WT mice with Aβo_1-42_ injections; Fig. 6D). Of note is that WT mice injected with Aβo_1-42_ did not significantly differ from WT mice with control injections, thus confirming an intermediate (sub-clinical) exposure to Aβo_1-42_. Neuronal count in a region distant to the injection site (i.e., CA2-CA3) revealed no significant difference between the four groups (Supplementary Fig. 8). These results suggest that the absence of NLGN1 potentiates the toxicity of Aβo_1-42_, leading to a noticeable neuronal loss in the DG.

## Discussion

Our findings provide insight on the involvement of NLGN1 in neurodegeneration. We first report that NLGN1 protein is decreased in the hippocampus of aMCI individuals and AD patients, in whom its level correlates with soluble Aβ and cognitive function. We then found that NLGN1 protein is also decreased, at an early developmental age, in the hippocampus of a mouse model showing Aβ pathology. Further, we revealed that the specific exposure to Aβo_1-42_ decreases NLGN1 protein in hippocampal neurons in culture and modulates *Nlgn1* hippocampal expression in a transcript variant-dependent manner. Importantly, we demonstrated a negative effect of the absence of NLGN1 on Aβo_1-42_ toxicity, with *Nlgn1* KO mice showing neuronal loss and greater deficits in spatial learning when injected with Aβo_1-42_ in comparison to WT animals. These results are supporting an impact of Aβo_1-42_ on NLGN1 as well as a modulatory role for NLGN1 in the toxicity induced by Aβo_1-42_.

To our knowledge, our study is the first to show that NLGN1 level in the hippocampus is decreased in aMCI individuals and AD patients. This result is in line with the recent report that NLGN1 is decreased in the plasma of AD patients and in individuals with less severe cognitive symptoms^39^, and may suggest that changes in the hippocampus could contribute to plasmatic changes. It also aligns with our previous findings of decreased synaptic proteins linked to glutamatergic transmission (e.g., PSD95, GluN2B) in the hippocampus of aMCI patients^53^. Our observation that the NLGN1 decrease is of greater magnitude than the PSD95 decrease emphasizes the particular relevance of considering the role of NLGN1 in neurodegeneration. Such a role is also supported by the observation of a NLGN1 mutation affecting its function in a patient with AD^35^. Moreover, the finding that NLGN1 level in the aMCI/AD hippocampus correlates with cognitive function supports the idea that synaptic dysfunction adequately predicts cognitive decline in AD^1–3,22^. Even if the exact mechanisms underlying NLGN modification in aMCI and AD remains to be identified, plasmatic NLGN1 or even hippocampal NLGN1 measured via imaging may be considered as an early biomarker of AD.

Importantly, we observed that the decrease in hippocampal NLGN1 protein is also an early event occurring in a recognized mouse model of AD (i.e., 3xTg-AD). Indeed, decreased NLGN1 was found at 4 months, an age at which cognitive symptoms are just starting to manifest and have been attributed to intraneuronal Aβ^57^. This model generally shows signs of neuronal death, human Aβ precursor protein (hAPP) deregulation, and Tau pathology later in life^42,60,61^, but these may all contribute to NLGN1 decrease together with alterations in Preselinin-1 in this model. The observation that only the females show this early decrease likely resides in the worst pathology observed in 3xTg-AD females in comparison to 3xTg-AD males. Indeed, 3xTg-AD females have repeatedly been reported to have, in particular, higher hAPP and Aβ levels than males^60,62,63^. Alternatively, a compensatory mechanism linked to the encoding of other NLGNs by sex chromosomes^64,65^, could also contribute to the sex-dependent decrease in NLGN1. Of interest is also that we found a genotype-independent decrease in NLGN1 level with age in male mice (also significant when females and males are pooled). This age-dependent decrease in hippocampal NLGN1 could definitely contribute to the main risk factor for neurodegeneration, namely age. More precisely, a progressive decrease in NLGN1 in the hippocampus may increase the susceptibility of this key brain region to synaptic and cell loss, such as under exposure to Aβo.

Our discovery that NLGN1 level in the hippocampus of aMCI individuals and AD patients significantly correlates with soluble Aβ could suggest a specific contribution of the Aβ pathology in decreasing NLGN1 level. We indeed specifically found that exposure to Aβo_1-42_ reduces NLGN1 level in cultured hippocampal neurons, similar to a recent study^66^. From these data, we cannot exclude a direct effect of neuronal death on NLGN1 level because the NLGN1 decrease paralleled the neuronal viability decrease. Still, these findings are revealing a synaptic component linked to the established neurotoxicity of Aβo as well as their correlation with cognitive functioning in AD patients and animal models^9–18^, given the roles of NLGN1 in cognition and its cellular correlates^3,31–33,64^. Of note is that Aβ_1-40_ fibrils, which are generally considered less neurotoxic than Aβo^11,19^, have also been shown to negatively impact NLGN1 protein level in the rat hippocampus^36^. The precise manner by which Aβo are decreasing the level of NLGN1, as well as the contribution of neuronal death, remains a question of particular relevance to evaluate the potential for NLGN1 to become a therapeutic target. Previous reports have indicated that Aβo are directly interacting with NLGN1 *in vitro*^37,38^. Such a direct interaction may contribute to an increased NLGN1 protein degradation. Alternatively, the Aβo-induced NLGN1 decrease could result from changes in *Nlgn1* gene expression linked to epigenetic modifications such as what has been observed for Aβ fibrils^36^.

An impact of Aβo on *Nlgn1* gene expression is indeed supported by our findings in chronic hippocampal injections. Importantly, the different durations of Aβo treatment we have used allowed for the observation of a bi-phasic time course comprising an initial increase followed by a decrease. Moreover, this expression pattern was found only for specific *Nlgn1* variants and not for other variants or other related genes (i.e, *Nlgn2*, *Nrxns*). In fact, the initial increase was observed for *Nlgn1* without insert in splice site A, and the following decrease for *Nlgn1* without insert in sites A and B. These changes were substantial enough to be also captured by the common probe, but strongly suggest that the expression of *Nlgn1* without insert is primarily affected by Aβo. In contrast with other isoforms, the isoform of NLGN1 not containing any insert is located equally at glutamatergic and gamma-aminobutyric acid (GABA) synapses^67^. This could suggest that the negative impact on NLGN1 could play a role in GABAergic synapse dysfunctions observed in AD and animal models^68,69^, as well as in glutamatergic synapse modifications^8,12,21^. Besides, the initial increase in *Nlgn1* expression after 2 days of Aβo exposure might belong to a neuroprotective pathway similar to other pathways driven by Aβ in the hippocampus^70^. NLGN1 was already shown to protect against oxidative stress in a non-mammalian model^71^ and to express neuroprotective functions in hippocampal neurons^38,72^. Alternatively, lower *Nlgn1* expression at 4 days of Aβo exposure may result from an increased expression with the vehicle injection, which could also be linked to a neuroprotective role of NLGN1 in response simply to intra-hippocampal injections. This effect would again be very specific to *Nlgn1* lacking any insert and would be counteracted by Aβo exposure. The fact that a modulation by chronic Aβo hippocampal injections could not be detected at the protein level likely results from the absence of an isoform-specific antibody. Indeed, a change in NLGN1 isoform without insert could be masked by the absence of change in NLGN1 with insert B, which transcript is highly expressed in the hippocampus^67^.

In the second part of our study, we provide data supporting a modulatory role of NLGN1 on spatial learning deficit and neuronal loss mediated by exposure of the hippocampus to Aβo_1-42_. Firstly, *Nlgn1* KO mice injected with Aβo_1-42_ were the only group showing memory impairment in SOR. A similar finding was made in the MWM training phase, during which *Nlgn1* KO mice injected with Aβo_1-42_ showed a longer overall latency to reach the platform. Deficits have been observed in KO mice with a lower exposure/dose than those generally inducing impairments in SOR and the MWM task in WT animals^52,73,74^. *Nlgn1* KO mice were previously shown to have only a very subtle deficit in the MWM and to have a preserved locomotion^33^, which are in line with our observations of very few deficits in vehicle-injected KO mice. The fact that KO mice injected with Aβo_1-42_ were impaired specifically in the MWM training phase, whereas *Nlgn1* KO mice are not^33^, also emphasizes a more specific implication of NLGN1 in modulating Aβo-induced deficits in learning or in memory acquisition. Of note is that this effect of NLGN1 seems restricted to spatial memory given that working memory assessed using the Y-maze was not affected by chronic hippocampal injections of Aβo. Although a longer exposure of the hippocampus to Aβo was shown to impair memory using a similar task^12^, the lack of effect in the current study is likely resulting from working memory involving to a great extent the cerebral cortex^75,76^. The manner by which NLGN1 modulates spatial learning deficits mediated by Aβo will need to be determined in future studies. Yet, the literature could point to mechanisms related to direct physical interactions between NLGN1 and Aβo^37,38^ potentially sequestering toxic oligomers, or linked to NLGN1 cleavage that occurs in response to sustained synaptic activity^72^.

Secondly, neuronal count in the DG was decreased only in *Nlgn1* KO mice. The absence of NLGN1 thus seems to increase the neurotoxicity of Aβo by inducing neuronal loss following an intermediate exposure (i.e., 4 days) whereas neuronal loss is generally obtained from longer exposure (i.e., 6 days) or higher doses^12,73,74^, similar to memory deficits. It should be noted, however, that the NeuN staining used here is not a direct indication of neuronal death, but that other accepted markers of neurodegeneration such as Caspase-3 and Fluoro-Jade B^12,77^ could not be used in the present study due to the sacrifice of animals being performed multiple days after exposure to allow for the evaluation of memory. Nevertheless, our finding supporting that NLGN1 can oppose Aβo-driven neurotoxicity in the DG is in line with observations that a soluble fragment of NLGN1 diminishes the negative impact of Aβo on excitatory synaptic transmission in hippocampal slices^38^ and that NLGN1 is part of a pathway counteracting Aβo-induced synaptotoxicity in cultured hippocampal neurons^66^.

## Conclusions

We here reveal that NLGN1 is decreased in the hippocampus of patients with prodromal signs of and established neurodegeneration in a symptomatology-dependent manner and, using *in vitro* and *in vivo* models, that this change is likely driven, at least in part, by Aβo and contributing to cognitive decline. In parallel, our data expose a bi-directional relationship between NLGN1 and Aβo, in which the accumulation of Aβo_1-42_ negatively impacts NLGN1, and decreased NLGN1 renders neurons/synapses more vulnerable to Aβo_1-42_ translating in memory deficits and neuronal loss. Our findings support a potential for strategies enhancing NLGN1 to alleviate symptoms of AD, which reconciles with previous research^36,38,78^, including with that reporting NRXN-NLGN mutations in AD patients^34,35^. On the contrary, our study does not support approaches opposing NLGN1 as a therapeutic strategy in AD such as the use of anti-NLGN1 antibody^37^. The current research provides additional insight in the development of early diagnostic tools and treatments of aMCI and AD, which is of crucial importance to benefit the quality of life of millions of AD patients worldwide.

## Supplementary information

Supplementary Fig. 1 to 8.
