# Supplementary material for: Neuroligin-1 is altered in the hippocampus of Alzheimer’s disease patients and mouse models, and modulates the toxicity of amyloid-beta oligomers
Source: Sci Rep. 2020 Apr 24;10:6956. doi: 10.1038/s41598-020-63255-6 (PMC7181681; doi:10.1038/s41598-020-63255-6)
Supplement: Supplementary file 1 — Supplementary Fig. 1 to 8. [file 41598_2020_63255_MOESM1_ESM.pdf]

# Neuroligin-1 is altered in the hippocampus of Alzheimer's disease patients and mouse models, and modulates the toxicity of amyloid-beta oligomers - SUPPLEMENTARY INFORMATION

**Julien Dufort-Gervais[1,2], Chloé Provost[2], Laurence Charbonneau[3], Christopher M. Norris[4], Frédéric Calon[5,6], Valérie Mongrain[2,3,7,*], Jonathan Brouillette[1,2,7,*]**

[1]Department of Pharmacology and Physiology, Université de Montréal, Montréal, Québec, Canada

[2]Center for Advanced Research in Sleep Medicine, Hôpital du Sacré-Coeur de Montréal (Recherche CIUSSS-NIM), Montréal, Québec, Canada

[3]Department of Neuroscience, Université de Montréal, Montréal, Québec, Canada

[4]Department of Molecular and Biomedical Pharmacology, Sanders-Brown Center on Aging, University of Kentucky, Lexington, KY, USA

[5]Neuroscience Unit, Research Center - CHU de Québec, Québec, QC, Canada

[6]Faculty of Pharmacy, Université Laval, Québec, QC, Canada

[7]These authors contributed equally to this work

***Corresponding authors:**

Valérie Mongrain: valerie.mongrain@umontreal.ca

Jonathan Brouillette: jonathan.brouillette@umontreal.ca

Recherche CIUSSS-NIM (site Hôpital du Sacré-Coeur de Montréal)

5400 Gouin West blvd.

Montreal, QC, H4J 1C5

Canada

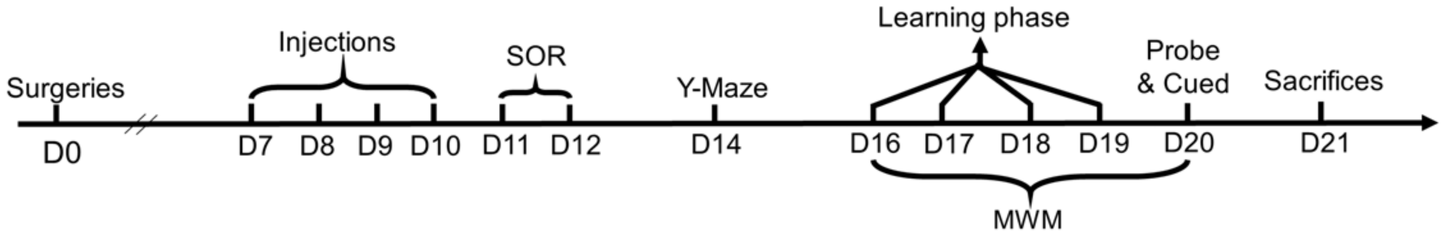


**Supplementary Fig. 1 -** Timeline of the experimental design for memory tests. Seven days after cannula implantation surgery, four consecutive days of hippocampal Aβo injections were performed, followed by the spatial object recognition (SOR) test (performed over 2 days), the Y-maze and the Morris water maze (MWM; performed over 5 days). Mice were sacrificed to perform hippocampal neuronal counts 24 hours after the last day of the MWM.

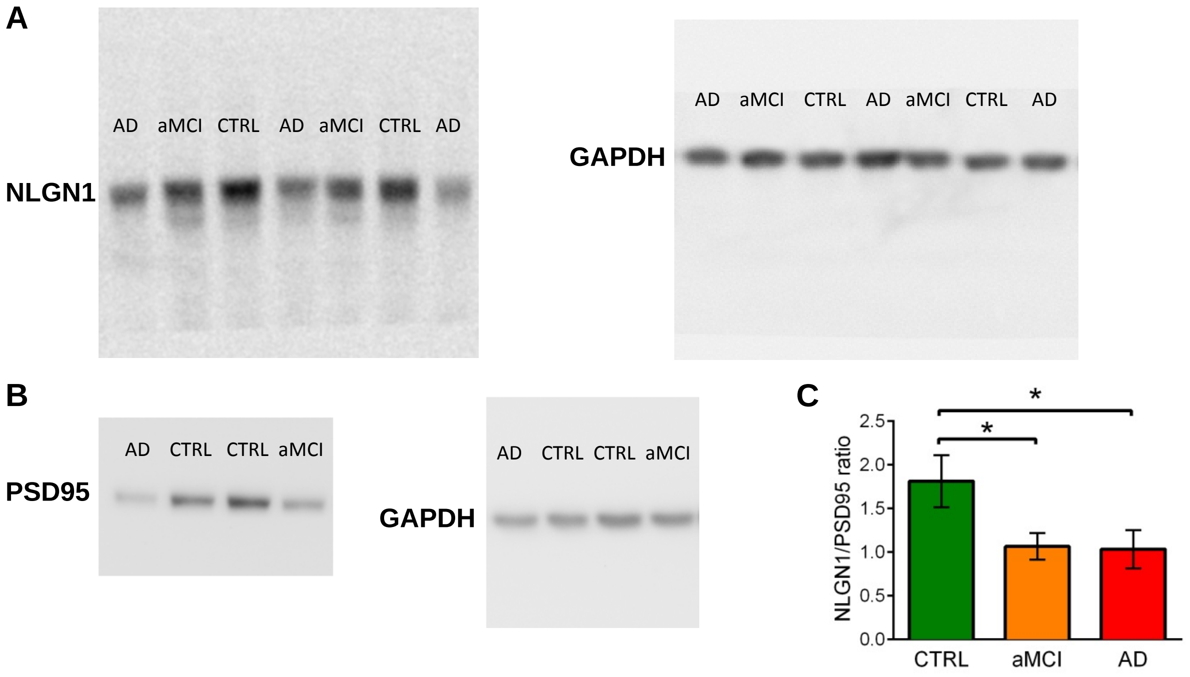


**Supplementary Fig. 2 - (A)** Full length blots of selected bands shown in Fig. 1A. Left blots correspond to NLGN1 (Millipore, Cat No. MABN742, 1:1000), right blots to GAPDH (Cell Signaling Technology, Cat No. 5174, 1:1000). Membranes were cut to separately blot for NLGN1 and GAPDH (same for other supplementary figures). Blots were run in parallel and imaged simultaneously. **(B)** Full length blots of representative bands corresponding to PSD95 (left: Millipore, Cat No. 04-1066, 1:1500) and GAPDH (right). **(C)** Quantification of the NLGN1 to PSD95 ratio (using GAPDH as a loading control for both synaptic proteins). Significant differences are represented between indicated groups (one-way ANOVA $F_{2,33} = 3.6$; $p = 0.04$; stars indicating planned comparisons $p < 0.05$). CTRL = healthy controls, aMCI = amnesic mild cognitive impairment, AD = Alzheimer's disease.

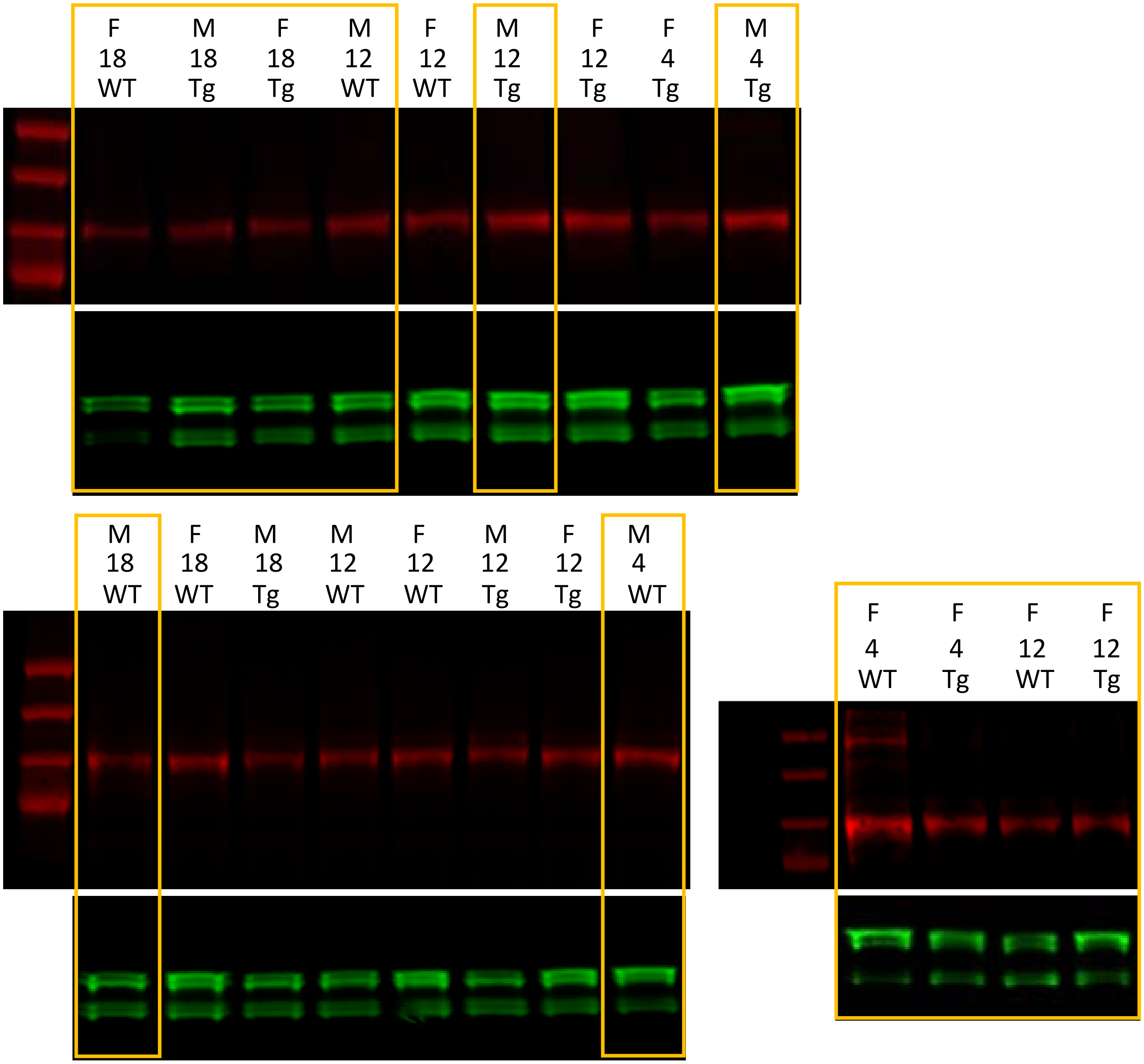


**Supplementary Fig. 3 -** Full length blots of selected bands shown in Fig. 2 (bands specifically featured in Fig. 2 are identified by yellow boxes). Red bands correspond to NLGN1 (Synaptic Systems, Cat No. 129 111, 1:1000) and green bands to GAPDH (Cell Signaling Technology, Cat No. 5174, 1:1000). Blots were run in parallel and imaged simultaneously. F: female; M: male; 4: 4 months; 12: 12 months; 18: 18 months; WT: wild-type; Tg: 3xTg-AD.

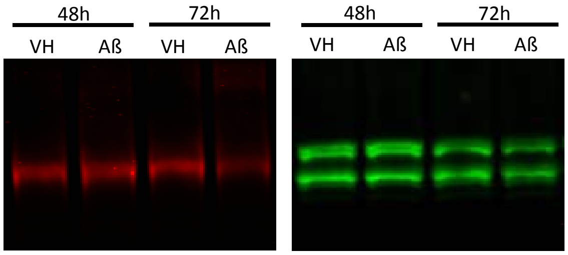


**Supplementary Fig. 4 -** Full length blots of selected bands shown in Fig. 3B. Red bands correspond to NLGN1 (Synaptic Systems, Cat No. 129 111, 1:1000) and green bands to GAPDH (Cell Signaling Technology, Cat No. 5174, 1:1000). Blots were run in parallel and imaged simultaneously.

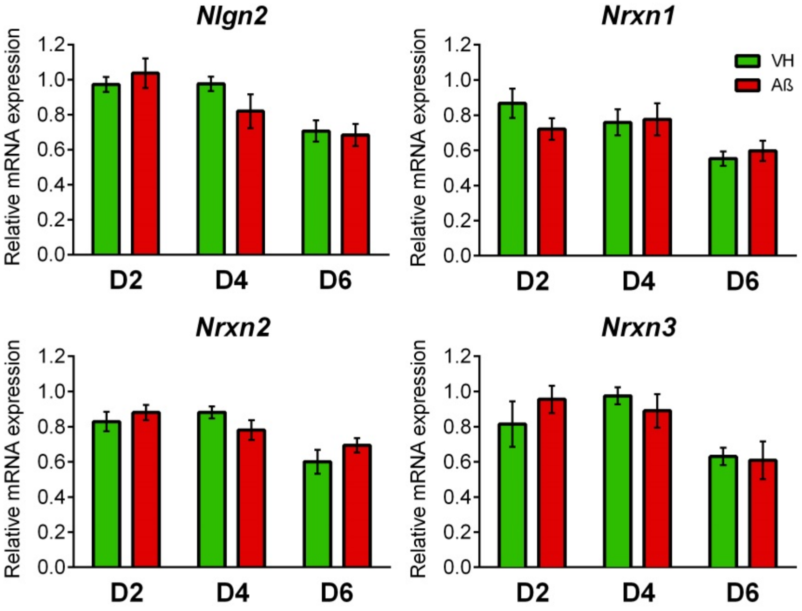


**Supplementary Fig. 5 -** Relative mRNA expression of Neuroligin-2 (*Nlgn2*), Neurexin-1 (*Nrxn1*), Neurexin-2 (*Nrxn2*) and Neurexin-3 (*Nrxn3*) after 2, 4 or 6 days of chronic hippocampal injections of Aßo$_{1-42}$ in mice. No significant effect of Treatment or Treatment-by-Day interaction was found for these four genes (*Nlgn2* Treatment: $F_{1,30} = 0.5$ p = 0.5; interaction: $F_{2,30} = 1.4$, p = 0.3; *Nrxn1* Treatment: $F_{1,30} = 0.2$ p = 0.6; interaction: $F_{2,30} = 1.1$, p = 0.4; *Nrxn2* Treatment: $F_{1,30} = 0.1$ p = 0.7; interaction: $F_{2,30} = 2.0$, p = 0.1; *Nrxn3* Treatment: $F_{1,30} = 0.02$ p = 0.9; interaction: $F_{2,30} = 0.8$, p = 0.4).

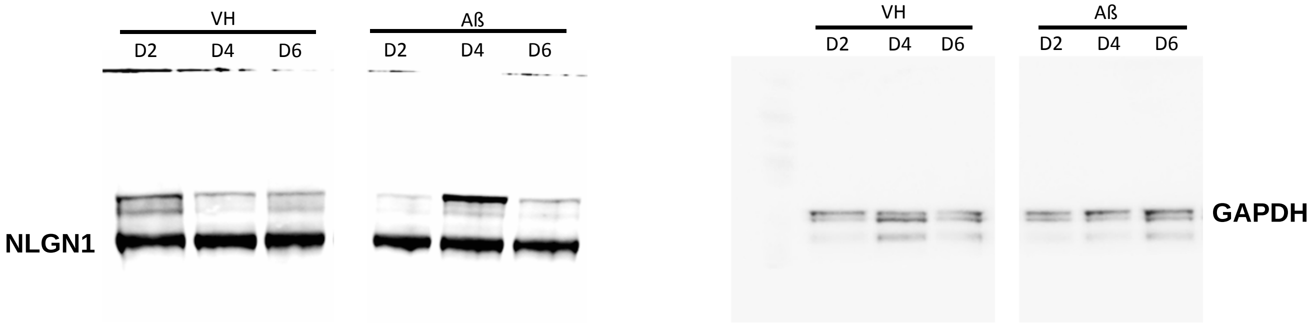


**Supplementary Fig. 6** - Full length blots of selected bands shown in Fig. 4F. Left blots are NLGN1 (Synaptic Systems, Cat No. 129 111, 1:1000), right blots are GAPDH(Cell Signaling Technology, Cat No. 5174, 1:1000). Blots were run in parallel and imaged simultaneously.

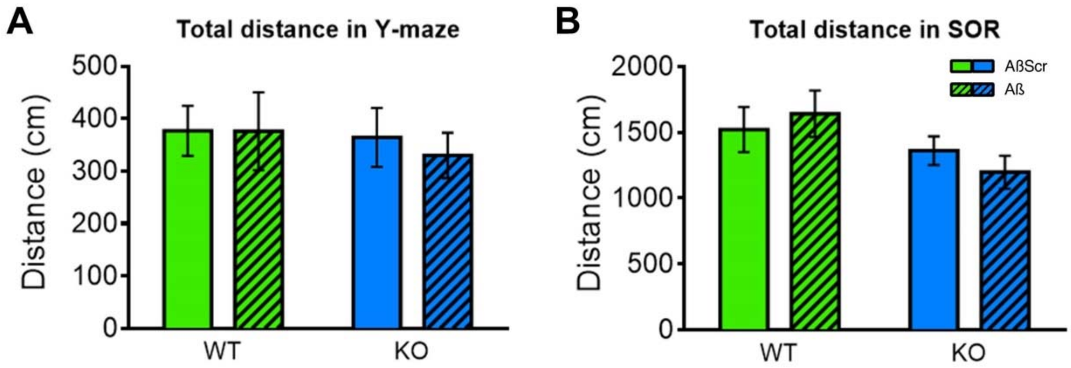


**Supplementary Fig. 7** – Total distance traveled in the Y-maze **(A)** and SOR **(B)** tests. All 4 groups traveled a similar distance during the Y-maze test (two-way ANOVA Genotype main effect: $F_{1,34} = 0.3$, $p = 0.6$; Treatment main effect: $F_{1,34} = 0.1$ $p = 0.7$; Genotype-by-Treatment interaction: $F_{1,34} = 0.1$, $p = 0.8$). A significant Genotype effect was found for the total distance traveled in the SOR test (Genotype effect: $F_{1,34} = 4.3$, $p = 0.046$; Treatment main effect: $F_{1,34} = 0.02$ $p = 0.9$; Genotype-by-Treatment interaction: $F_{1,34} = 0.9$, $p = 0.3$). The number of mice is 9-10 per group.

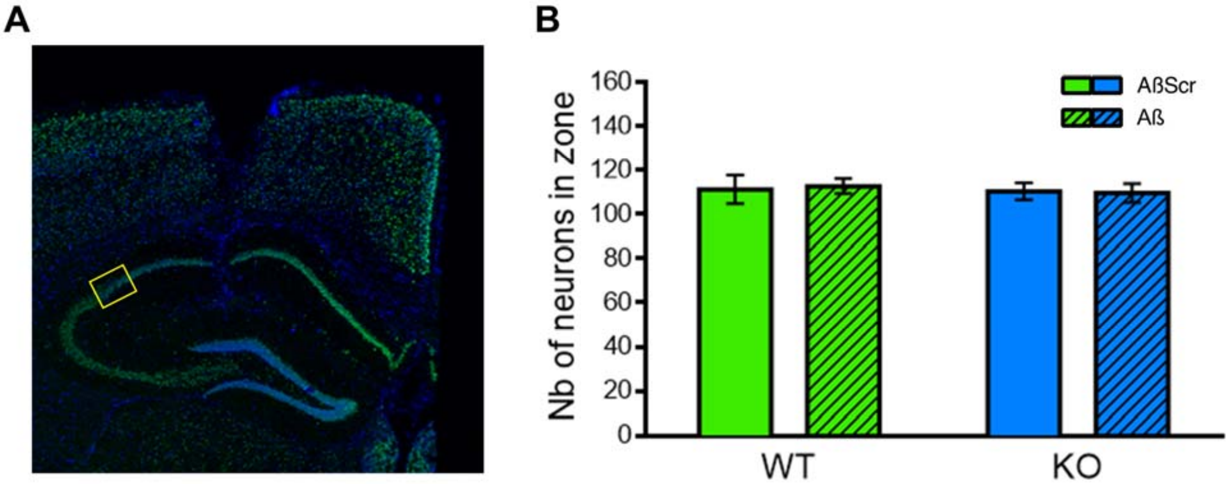


**Supplementary Fig. 8 - (A)** Representative IF image of NeuN staining (green) in the full hippocampus. The counting zone distant to the injection site is identified with a yellow rectangle. Magnification 2.5X **(B)** The number of NeuN-positive cells counted in the CA2-CA3 region distant to the injection site. No significant difference between the 4 groups was observed (two-way ANOVA Genotype main effect: $F_{1,20} = 0.2$, $p = 0.7$; Treatment main effect: $F_{1,20} = 0.006$ $p = 0.9$; Genotype-by-Treatment interaction: $F_{1,20} = 0.1$, $p = 0.8$). The number of mice is 6 per group.
